# Supplementary material for: Dysfunctional Sars-CoV-2-M protein-specific cytotoxic T lymphocytes in patients recovering from severe COVID-19
Source: Nat Commun. 2022 Dec 16;13:7063. doi: 10.1038/s41467-022-34655-1 (PMC9758236; doi:10.1038/s41467-022-34655-1)
Supplement: Supplementary file 1 — Supplementary Information [file 41467_2022_34655_MOESM1_ESM.pdf]

Supplementary Information

Dysfunctional Sars-CoV-2 M protein-specific cytotoxic T lymphocytes in patients recovering from severe COVID-19

Authors

Hideki Ogura[1*], Jin Gohda[2], Xiuyuan Lu[3], Mizuki Yamamoto[2], Yoshio Takesue[4,5], Aoi Son[1], Sadayuki Doi[6], Kazuyuki Matsushita[7], Fumitaka Isobe[8], Yoshihiro Fukuda[9], Tai-Ping Huang[10], Takamasa Ueno[11], Naomi Mambo[12], Hiromoto Murakami[12], Yasushi Kawaguchi[2,13], Jun-ichiro Inoue[14], Kunihiro Shirai[12], Sho Yamasaki[3,15,16,17], Jun-Ichi Hirata[12] and Satoshi Ishido[1*]

Affiliations

[1]Department of Microbiology, Hyogo Medical University, Hyogo, Japan.

[2]Research Center for Asian Infectious Disease, The Institute of Medical Science, The University of Tokyo, Tokyo, Japan.

[3]Laboratory of Molecular Immunology, Immunology Frontier Research Center, Osaka University, Suita, Japan.

[4]Department of Infection Control and Prevention, Hyogo Medical University, Hyogo, Japan.

[5]Tokoname City Hospital, Aichi, Japan.

[6]Kawanishi City Hospital, Hyogo, Japan.

[7]Kyoritsu Hospital, Hyogo, Japan.

[8]Kyowa Marina Hospital/Wellhouse Nishinomiya, Hyogo, Japan.

[9]Dainikyoritsu Hospital, Hyogo, Japan.

[10]Kyoritsu Onsen Hospital, Hyogo, Japan.

[11]Joint Research Center for Human Retrovirus Infection, Kumamoto University, Kumamoto, Japan.

[12]Department of Emergency and Critical Care Medicine, Hyogo Medical University, Hyogo, Japan.

[13]Division of Molecular Virology, Department of Microbiology and Immunology, The Institute of Medical Science, The University of Tokyo, Tokyo, Japan.

[14]Research Platform Office, The Institute of Medical Science, The University of Tokyo, Tokyo, Japan.

[15]Department of Molecular Immunology, Research Institute for Microbial Diseases, Osaka University, Suita, Japan.

[16]Division of Molecular Design, Medical Institute of Bioregulation, Kyushu University, Fukuoka, Japan.

[17]Division of Molecular Immunology, Medical Mycology Research Center, Chiba University, Chiba, Japan.

*Corresponding authors:

Hideki Ogura: hi-ogura@hyo-med.ac.jp

Satoshi Ishido: sh-ishido@hyo-med.ac.jp

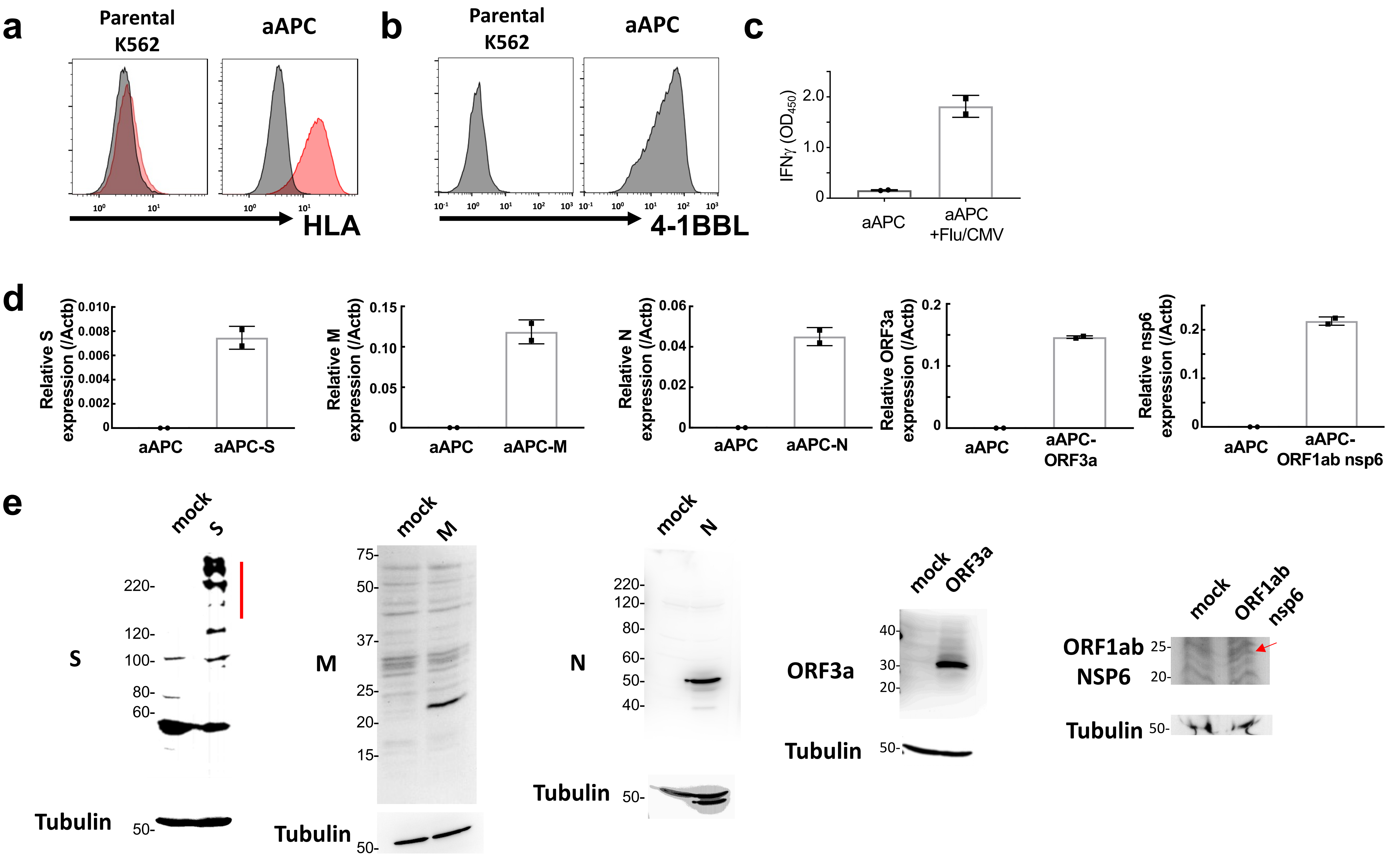

Supplementary Figure 1 | Preparation of viral-protein-expressing artificial antigen presenting cells (aAPC). K562 cells were transfected with HLA-A*24:02 and costimulatory molecule 4-1BBL overexpression vectors, then several viral genes (S, M, N, ORF3a, ORF1ab nsp6) were transduced individually. **a, b** pan HLA **(a)** or costimulatory molecule 4-1BBL **(b)** staining of aAPC and parental K562 cell line. **c** An influenza virus and cytomegalovirus (Flu/CMV)-peptide specific $CD8^+$ T cell line isolated from HLA-A*$24:02^+$ donor was cocultured with aAPC in the presence or absence of Flu/CMV-peptides (n=2, biologically independent samples). OD_{450} value was measured by IFNγ ELISA. Data represent mean + SD. **d** mRNA expression level of viral genes (S, M, N, ORF3a, and ORF1ab nsp6) in aAPC were examined by real-time quantitative PCR (n=2, biologically independent samples). Data are presented as mean values +/- SD. **e** Protein expression level of viral genes (S, M, N, ORF3a, and ORF1ab nsp6) in aAPC were assessed by western blot using specific primers and antibodies, respectively.

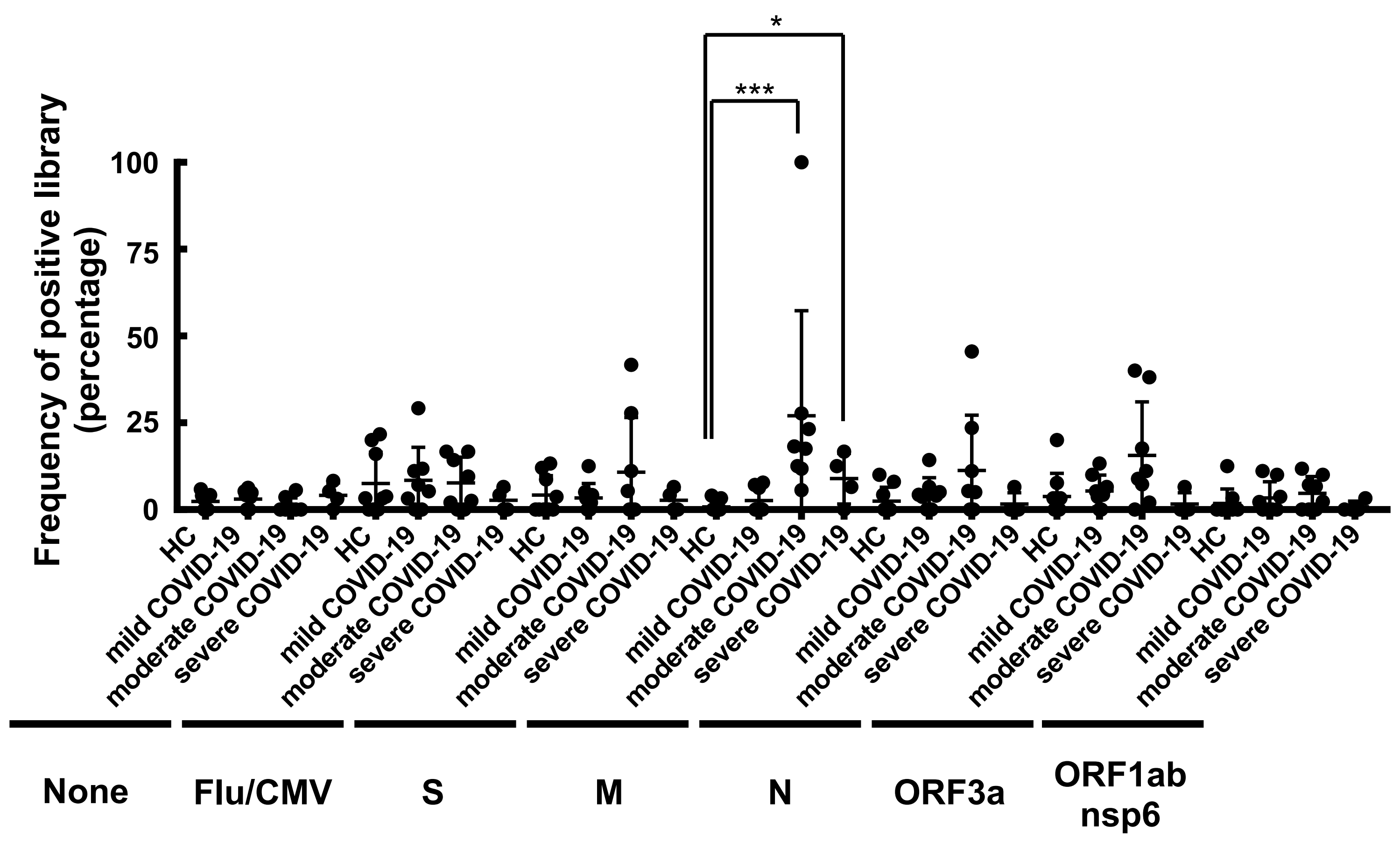

Supplementary Figure 2 | CD8⁺ T cell library assay results on convalescents recovered from COVID-19 with different severities. The frequencies of positive libraries are shown. Each dot represents each subject with bars showing mean and SD values (healthy donors n=8; mild, n=8; moderate, n=8; severe, n=4). p values were calculated by two-sided Mann-Whitney test. *p = 0.024; *** p = 0.0002.

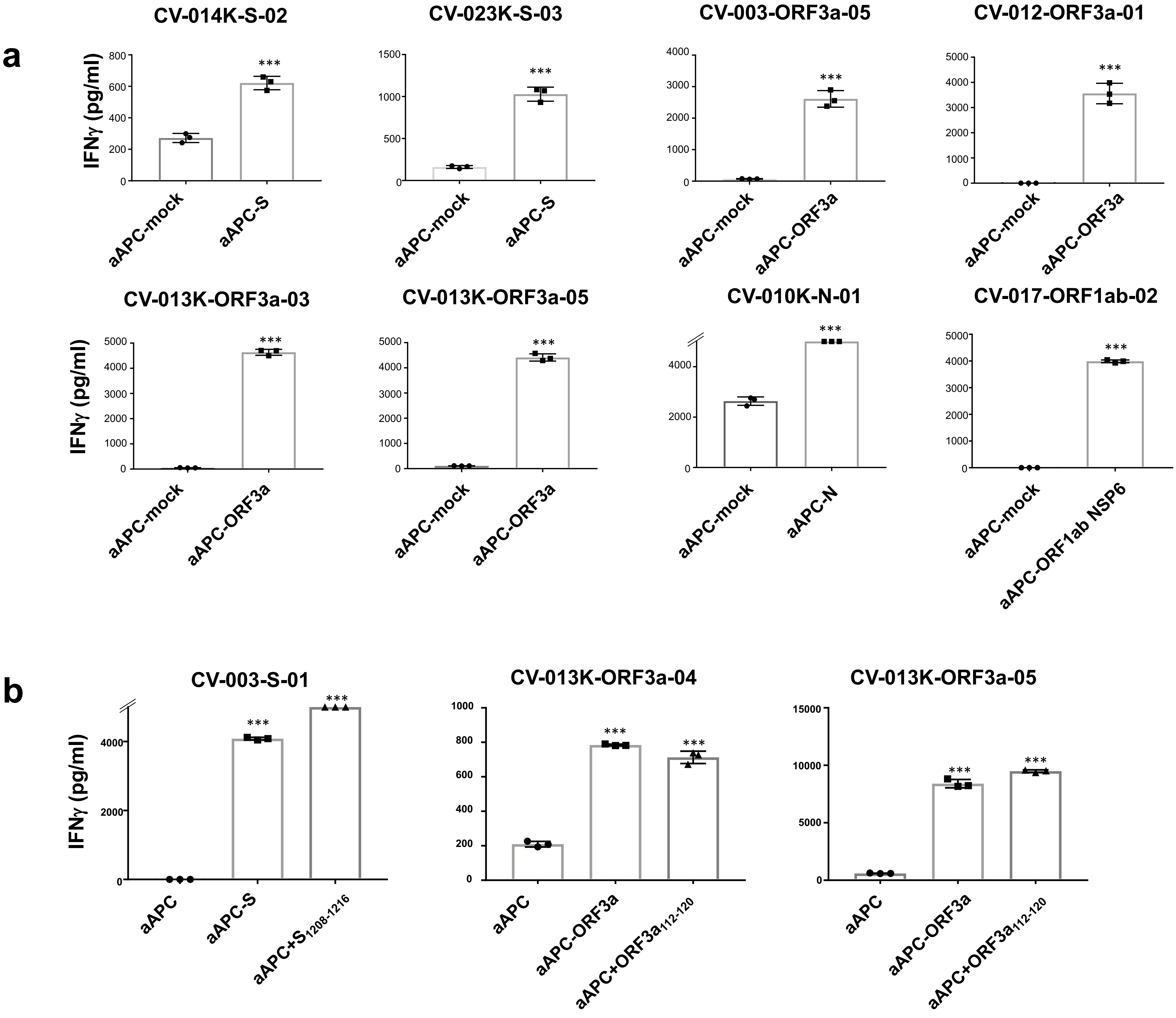

Supplementary Figure 3 | CD8⁺ T cell library assay with artificial antigen presenting cells identified broad spectrum of viral-antigen specific CD8⁺ T cells. a Examples of viral proteins-specific CD8⁺ T cells identified in the CD8⁺ T cell library assay. The libraries with positive response against viral protein-expressed artificial antigen presenting cells (aAPC) in CD8⁺ T cell library assay were further expanded and split into multiple wells. Then the cells were stimulated with either control aAPC or corresponding viral antigen-expressing aAPCs (aAPC-S, aAPC-ORF3a, aAPC-N, and aAPC-ORF1ab NSP6, individually). Supernatant were collected and subjected to IFNγ ELISA. Wells were triplicated and p values were calculated by two-sided unpaired t test. *** p = 0.0003 (CV-014K-S-02), 6.1×10^{-5} (CV-023K-S-03), 7.4×10^{-5} (CV-003-ORF3a-05), 0.00011 (CV-12-ORF3a-01), 3.0×10^{-7} (CV-013K-ORF3a-03), 8.6×10^{-7} (CV-013K-ORF3a-05), 1.6×10^{-5} (CV-010K-N-01), and 2.2×10^{-8} (CV-017-ORF1ab-02). Data are presented as mean values +/- SD. **b** Several viral protein-responding T cell libraries were screened for their specificity for reported immunodominant epitopes $S_{1208\text{-}1216}$ and $ORF3a_{112\text{-}120}$ by their IFNγ secretion ability. Wells were triplicated and p values were calculated by two-sided unpaired t test. *** $p = 5.3 \times 10^{-7}$, 3.7×10^{-8} (CV-003-S-01); *** $p = 4.8 \times 10^{-7}$, 2.4×10^{-5} (CV-013K-ORF3a-04); *** $p = 3.1 \times 10^{-6}$, 1.7×10^{-8} (CV-013K-ORF3a-05). Data are presented as mean values +/- SD. Results from representative T cell libraries are shown.

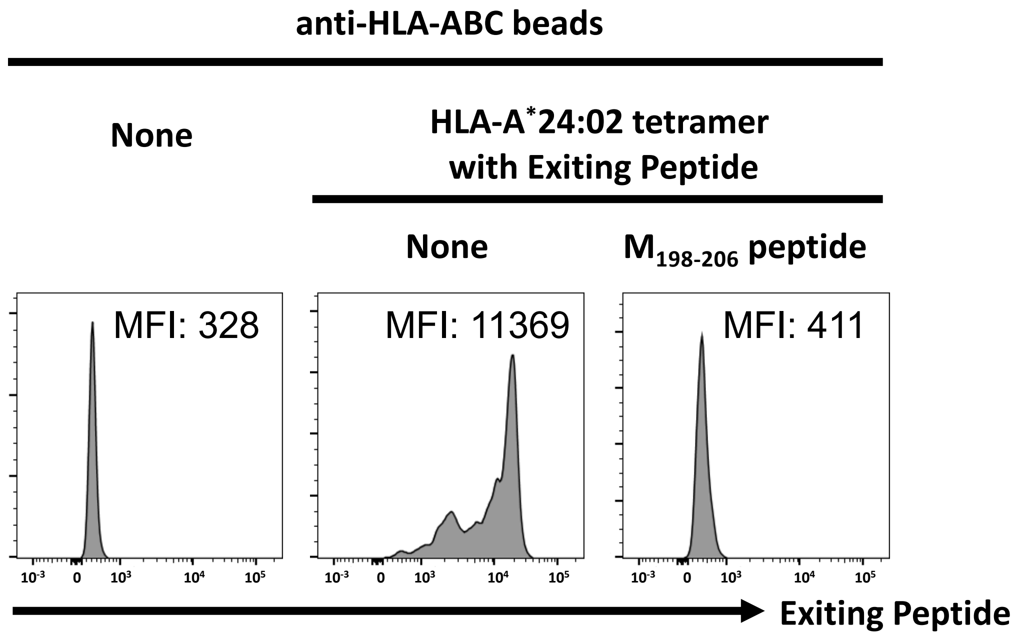

Supplementary Figure 4 | Direct association of $M_{198\text{-}206}$ peptide to HLA-A*24:02. HLA-A*24:02 tetramer with Exiting Peptide were incubated with or without $M_{198\text{-}206}$ peptide for 4 hours at room temperature. Then, anti-HLA-ABC beads were mixed with or without the tetramers and rinsed. The beads were stained with FITC-labeled anti-Exiting Peptide and subjected to flow cytometry analysis. Histograms are shown with MFI values in each panel. According to the MFI values, 99.2 % of Exiting Peptides on HLA-A*24:02 tetramer were replaced by $M_{198\text{-}206}$ peptide (see Methods).

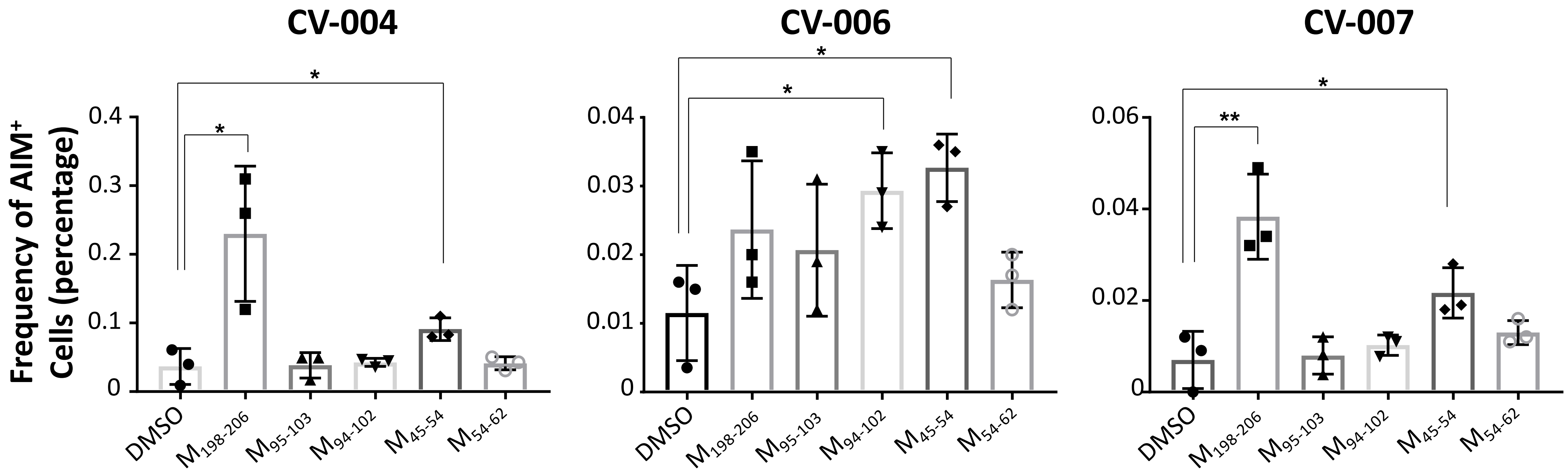

Supplementary Figure 5 | Activation induced marker assay. 1 x 10^6 PBMCs of indicated subjects (CV-004, CV-006, and CV-007) were incubated with candidate epitopes of SARS-CoV-2-M protein for 24 hours and then examined the expression of CD69 and CD137 by flow cytometry. Wells were triplicated. Graph indicates mean +/- SD and *p* values were calculated by two-sided unpaired t test. * p = 0.030 (DMSO-$M_{198\text{-}206}$), 0.038 (DMSO-$M_{45\text{-}54}$) for CV-004; * p = 0.025 (DMSO-$M_{94\text{-}102}$), 0.013 (DMSO-$M_{45\text{-}54}$) for CV-006; ** p = 0.0084 (DMSO-$M_{198\text{-}206}$), * p = 0.038 (DMSO-$M_{45\text{-}54}$) for CV-007.

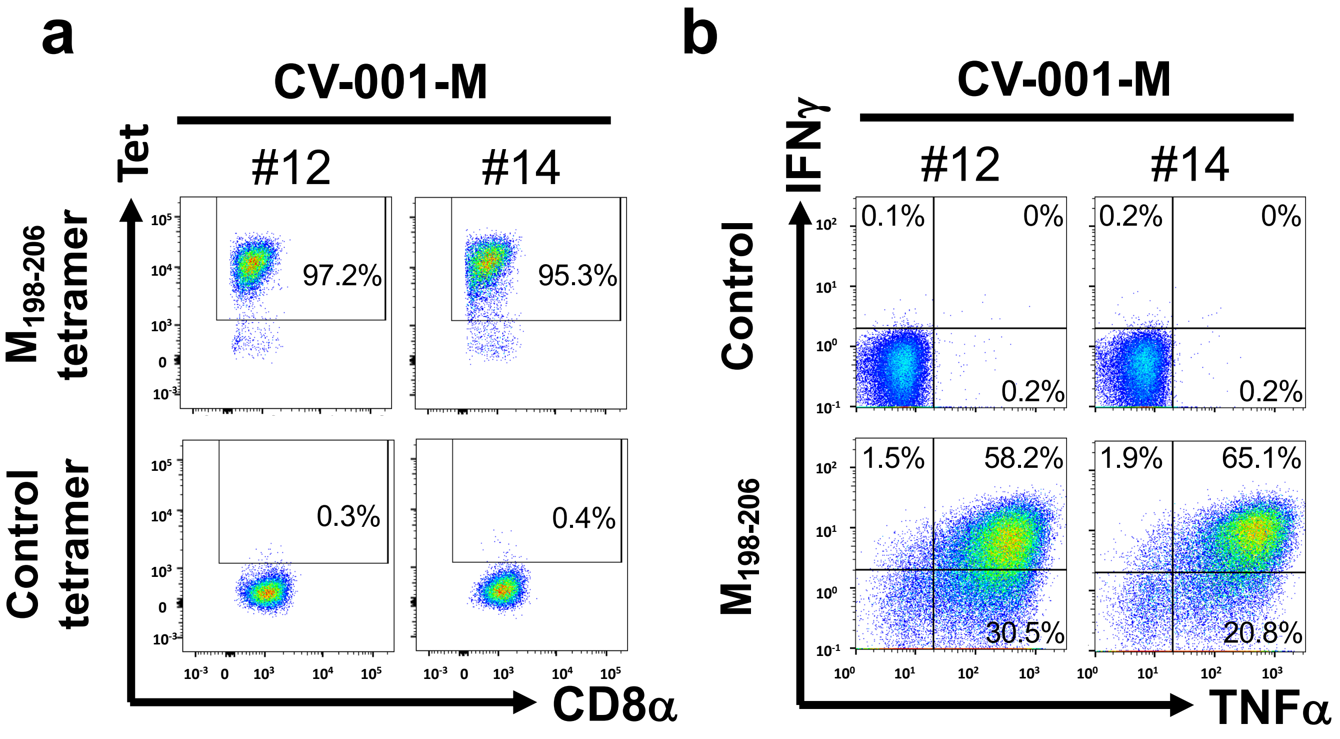

Supplementary Figure 6 | $M_{198-206}$ specific lines (CV-001-M-12 and CV-001-M-14) used in cytotoxic activity assay. M-responding libraries obtained by $CD8^+$ T cell library assay, CV-001-M-12 and CV-001-M-14, were further expanded and enriched (see Methods). Resulted cells were checked for their specificity for $M_{198-206}$ by MHC class I tetramer staining **(a)** and ICS **(b)**.

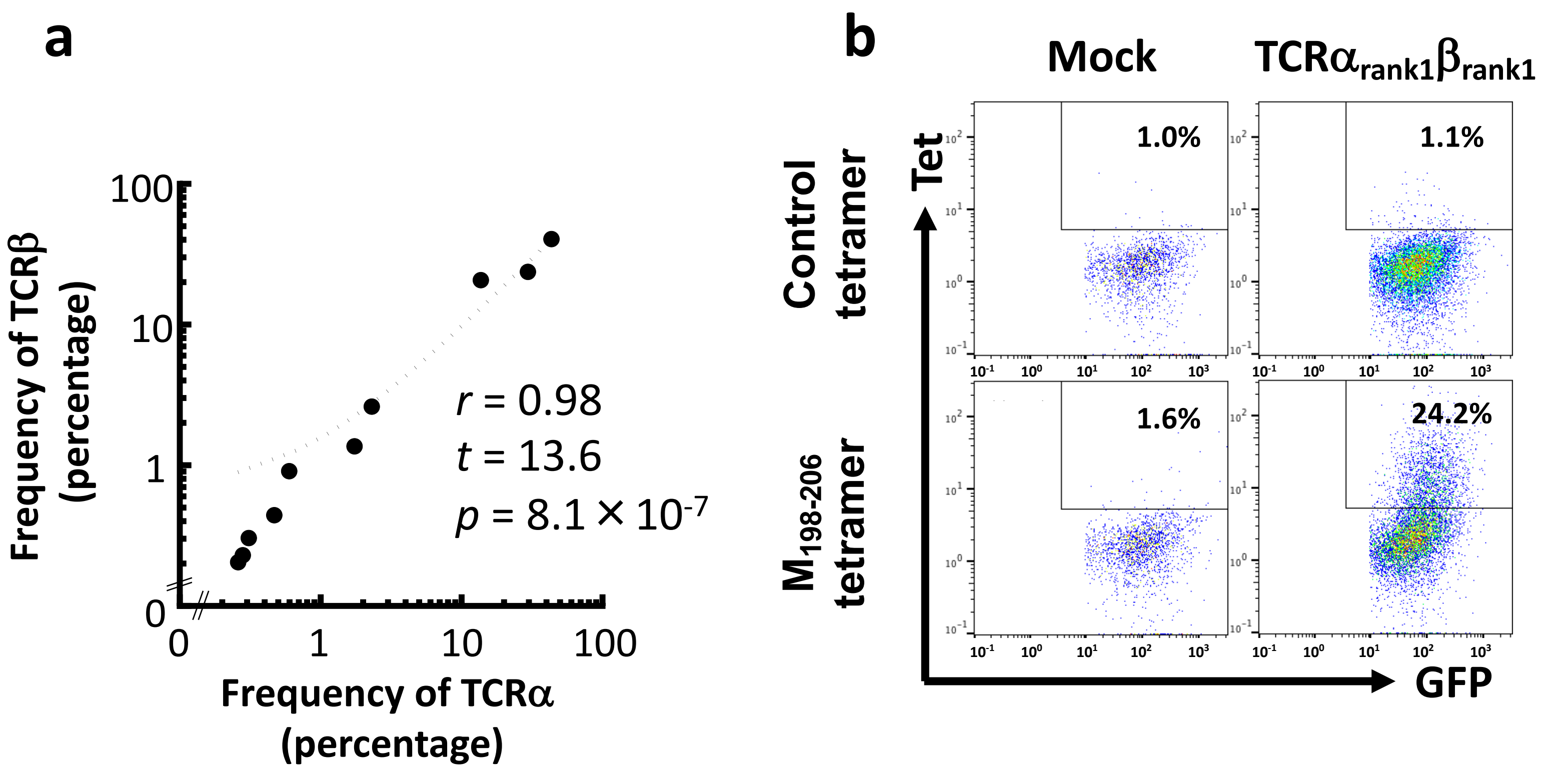

Supplementary Figure 7| SARS-CoV-2-$M_{198\text{-}206}$-specific TCR cloning. a Top 10 TCR alpha and beta sequences were paired and plotted based on their frequencies. Pearson *r* number, *t* value, and *p* value (two-tailed) are shown (r = 0.98, t = 13.6, $p = 8.1 \times 10^{-7}$). **b** Both rank1 chains of TCRα and β were co-expressed in TG40/CD8 cells and stained with $M_{198\text{-}206}$-tetramer.

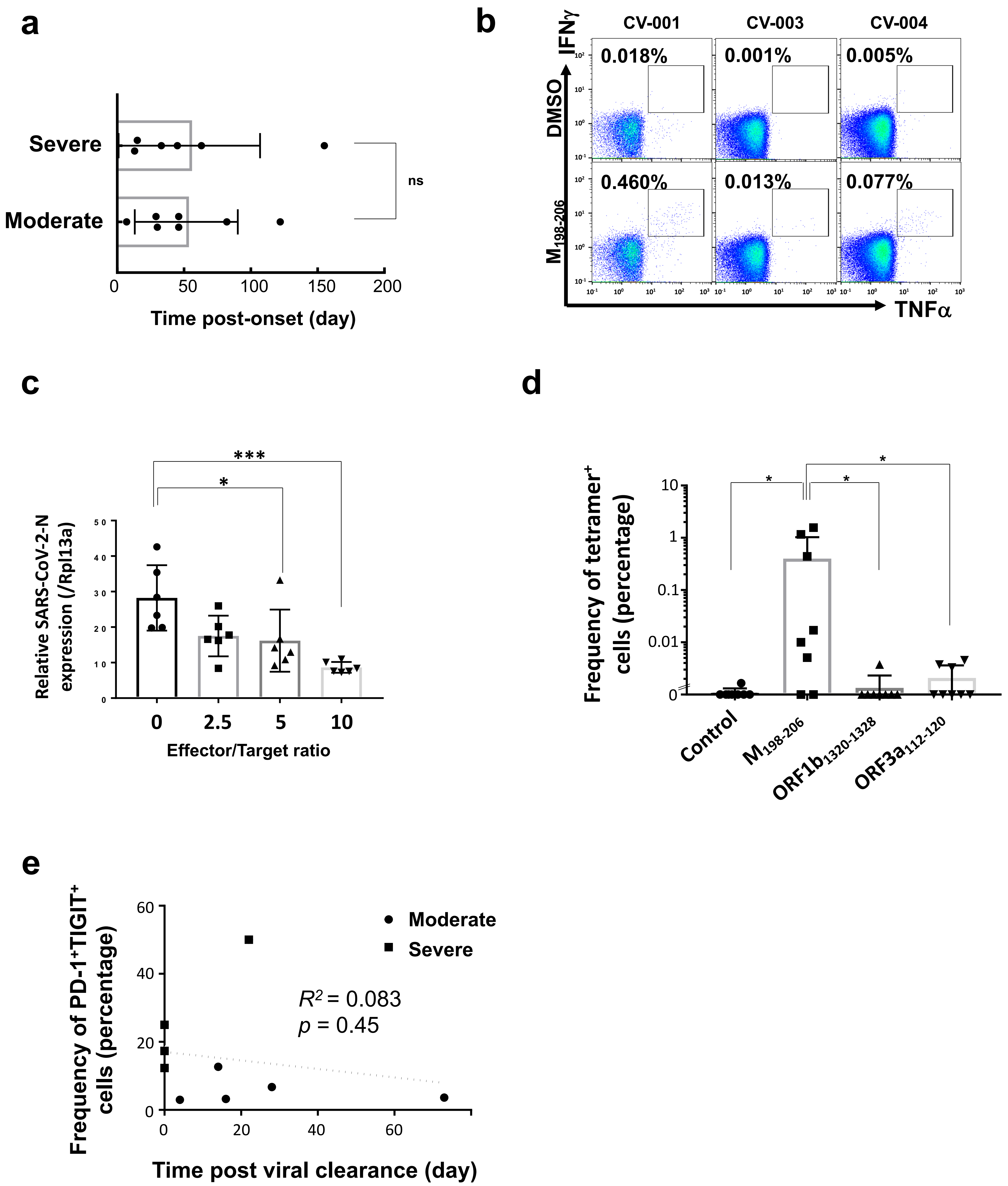

Supplementary Figure 8 | Significance of $M_{198\text{-}206}$ peptide. a Time post-onset of COVID-19 was shown in the bar graph. ns=not significant. p values were calculated by two-sided Mann-Whitney test. Data represent mean +/- SD. **b** PBMCs of indicated convalescents were incubated with $M_{198\text{-}206}$ peptide and ICS was performed. **c** Calu-3 cells (Target) were infected with SARS-CoV-2-Omicron and cocultured with a $M_{198\text{-}206}$-specific $CD8^+$ T cell (Effector) line derived from a T cell library. Calu-3 intracellular mRNA expression of SARS-CoV-2-N was assessed. Wells were sextupled and data represents mean + SD. *p* values were calculated by two-sided Mann-Whitney test. $^{*}p = 0.026$, *** p = 0.0022. **d** Frequency of $CD8^+$ T cells specific to $M_{198\text{-}206}$ and other epitopes ($ORF1b_{1320\text{-}1328}$, and $ORF3a_{112\text{-}120}$) of individual subjects were quantitated by flow cytometry using PE- and BV421-labeled MHC tetramer double staining. Data represent mean + SD and *p* values were calculated by two-sided Wilcoxon matched-pairs signed rank test. *p = 0.031 (Control-$M_{198\text{-}206}$), 0.031 ($M_{198\text{-}206}$-$ORF1b_{1320\text{-}1328}$), and 0.031($M_{198\text{-}206}$-$ORF3a_{112\text{-}120}$). **e** Correlation between frequency of $PD\text{-}1^+TIGIT^+$ cells among $tetramer^+CD8^+$ T cells and time post-clearance of the virus (moderate n=5; severe n=4). The non-parametric Spearman correlation coefficient and the *p* values (two-sided) were indicated in the panel.

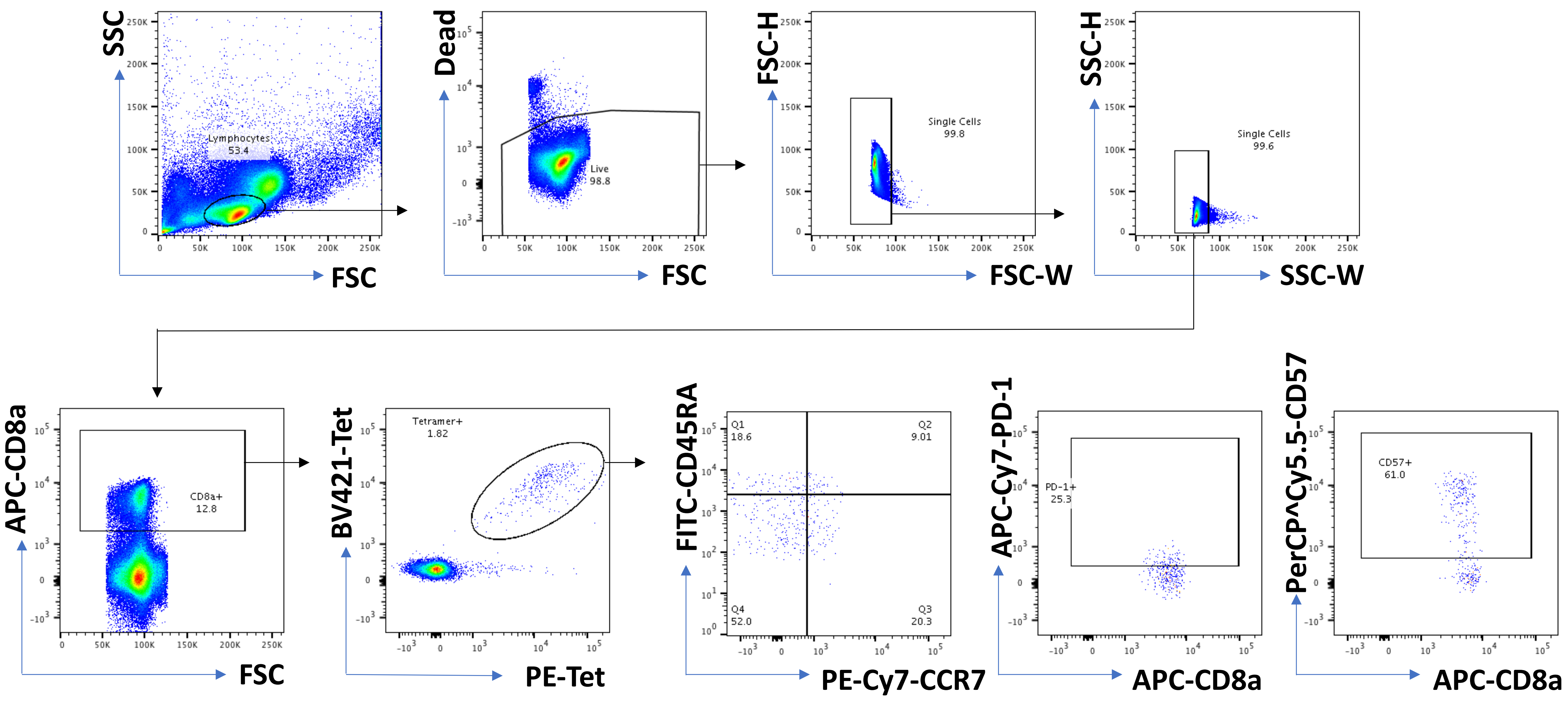

Supplementary Figure 9 | Gating strategy for identifying tetramer-positive cells and surface marker expression. Gating strategy to analyze tetramer⁺ cells.

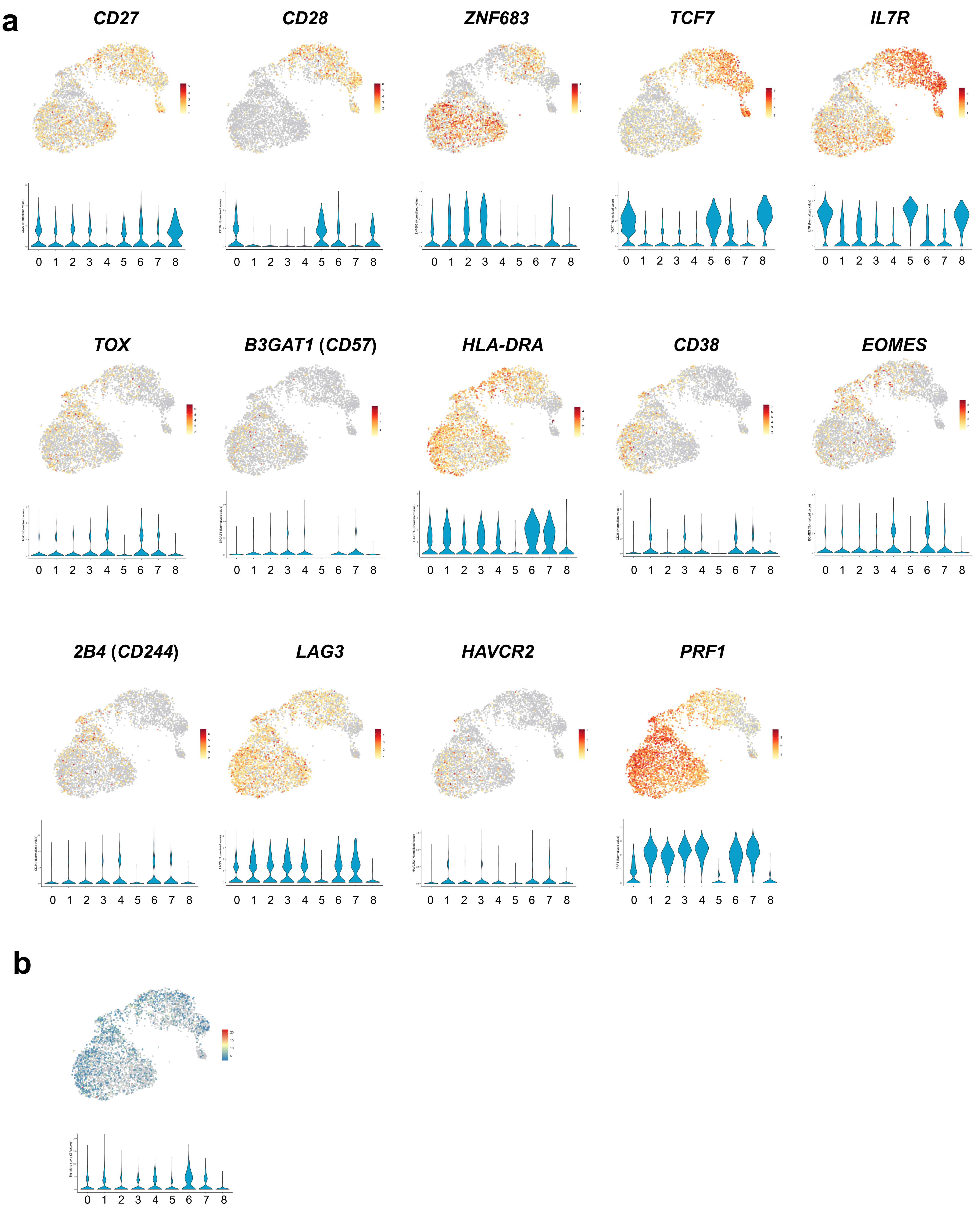

Supplementary Figure 10 | Gene signature of $M_{198\text{-}206}$-specific $CD8^{+}$ T cells. a mRNA expression of indicated genes were shown on UMAPs. The expression level on each cluster was shown in violin plot. **b** The exhaustion score signatured by mRNA expression of *PDCD1* and *TIGIT* was calculated and shown in color on UMAP and in violin plot.

Supplementary Table 1| Clinical characteristics of participants subjected to the study

ID	Gender	Age	Oxygen Supply	ECMO	History	Category	Library	Tetramer
HC-001	M	39	-	-		healthy	v	
HC-006	M	43	-	-		healthy	v	v
HC-007	F	20	-	-		healthy	v	v
HC-009	F	20	-	-		healthy	v	v
HC-010	F	20	-	-		healthy	v	v
HC-011	F	20	-	-		healthy	v	v
HC-012	F	20	-	-		healthy	v	
HC-013	M	48	-	-		healthy	v	
CV-001	M	77	2L/min	-	hypertension, hyperlipidemia, allergic rhinitis	moderate COVID-19	v	v
CV-002	M	68	4L/min	-		moderate COVID-19	v	v
CV-003	M	66	2L/min	-	T2DM	moderate COVID-19	v	v
CV-004	M	69	5L/min	-	hypertension	moderate COVID-19	v	v
CV-005	M	47	15L/min	+	T2DM, asthma	severe COVID-19	v	v
CV-006	M	56	2L/min	-	T2DM, chronic obstructive pulmonary disease	moderate COVID-19	v	v
CV-007	M	70	6L/min	-		moderate COVID-19	v	v
CV-008K	M	39	-	-		mild COVID-19	v	v
CV-009	M	71	15L/min	+	hypertension	severe COVID-19	v	v
CV-010K	F	51	-	-		mild COVID-19	v	v
CV-011K	M	35	-	-		mild COVID-19	v	v
CV-012	M	49	3L/min	-		moderate COVID-19	v	v
CV-013K	F	43	-	-		mild COVID-19	v	v
CV-014K	F	50	-	-		mild COVID-19	v	v
CV-017	M	33	3L/min	-		moderate COVID-19	v	v
CV-020K	F	50	-	-		mild COVID-19	v	v
CV-023K	M	58	-	-		mild COVID-19	v	v
CV-025K	F	47	-	-		mild COVID-19	v	v
CV-035	M	55	10L/min	+	pulmonary emphysema	severe COVID-19	v	v
CV-038	F	68	40L/min	+	hypertension, chronic obstructive pulmonary disease	severe COVID-19	v	v
HC-003	F	45	-	-		healthy		v
CV-026K	M	49	-	-		mild COVID-19		v
CV-031K	F	53	-	-		mild COVID-19		v
CV-032K	F	56	-	-		mild COVID-19		v
CV-034K	F	46	-	-		mild COVID-19		v
CV-037	F	70	10L/min	+	T2DM	severe COVID-19		v
CV-039	M	63	3L/min	+	hypertension	severe COVID-19		v
CV-041	F	68	8L/min	+	hypertension	severe COVID-19		v
CV-045	M	49	3L/min	+		severe COVID-19		v
CV-046	M	48	1L/min	+	T2DM, Kidney cyst	severe COVID-19		v
CV-048	M	39	-	+	hypertension, T2DM	severe COVID-19		v
CV-052	M	70	40L/min	+	hypertension	severe COVID-19		v
CV-057	M	69	38L/min	+	hypertension, Behcet's disease	severe COVID-19		v
CV-062	M	27	3L/min	-	hypertension	moderate COVID-19		v
CV-065	M	54	2L/min	-		moderate COVID-19		v
CV-071	M	63	10L/min	+	T2DM, diabetic nephropathy, arteriosclerosis obliterans	severe COVID-19		v
CV-073	M	65	9L/min	-		moderate COVID-19		v

COVID-19 convalescents were categorized into three groups (i.e., mild, moderate and severe) based on the extent of oxygen supplementation and requirement of mechanical ventilation (mild: no oxygen supplementation, moderate: oxygen supplementation $FiO_2 < 0.5$, severe: heavy oxygen supplementation $FiO_2 > 0.5$ and/or mechanical ventilation)

Supplementary Table 2| TCR α and β repertoire analysis

Rank	TRAV	TRAJ	CDR3	Reads	%Reads	Rank	TRBV	TRBJ	CDR3	Reads	%Reads
1	TRAV12-2	TRAJ23	CAVTNQGGKLIF	55238	43.4418107	1	TRBV3-1	TRBJ2-7	CASSQEGIEQYF	35386	40.549584
2	TRAV12-2	TRAJ37	CAVASGNTGKLIF	37767	29.7017789	2	TRBV27	TRBJ2-2	CASSLETGGTGELFF	20714	23.7366214
3	TRAV12-2	TRAJ29	CASLRGPLVF	17467	13.736886	3	TRBV3-1	TRBJ2-1	CASSEQGVEQFF	18059	20.6941993
4	TRAV1-2	TRAJ23	CAVIYNQGGKLIF	2938	2.31058402	4	TRBV29-1	TRBJ1-4	CSAFGTTNEKLFF	2278	2.61040955
5	TRAV12-3	TRAJ37	CASKGNTGKLIF	2208	1.73647703	5	TRBV7-8	TRBJ1-2	CASSAGGFGYGYTF	1192	1.36593862
6	TRAV17	TRAJ28	CATDAKPGAGSYQLTF	763	0.60005977	6	TRBV12-5	TRBJ1-1	CASGGDLTRGLYTEAFF	794	0.9098618
7	TRAV5	TRAJ5	CAEDSDTGRRALTF	599	0.47108231	7	TRBV6-4	TRBJ1-2	CASSDSGTDGYTF	387	0.44347168
8	TRAV30	TRAJ45	CGTGPPSRGGADGLTF	399	0.31379272	8	TRBV6-3	TRBJ2-1	CASSTDRRNYNEQFF	265	0.30366924
9	TRAV1-2	TRAJ23	CAVVYNQGGKLIF	356	0.27997546	9	TRBV13	TRBJ2-2	CASSSDRAPTGELFF	200	0.22918433
10	TRAV16	TRAJ34	CALGVSYNTDKLIF	325	0.25559558	10	TRBV6-4	TRBJ1-2	CASSDSGADGYTF	179	0.20511998
11	TRAV12-2	TRAJ45	CALGGGADGLTF	281	0.22099187	11	TRBV20-1	TRBJ2-7	CSGTSGTHSYEQYF	156	0.17876378
12	TRAV3	TRAJ21	CAVRDLDNFNKFYF	211	0.16594051	12	TRBV27	TRBJ2-3	CASSFRSTDTQYF	150	0.17188825
13	TRAV20	TRAJ36	CAVQADRTGANNLFF	193	0.15178445	13	TRBV3-1	TRBJ2-1	CASSQEGIEQYF	137	0.15699127
14	TRAV12-3	TRAJ23	CAVTNQGGKLIF	191	0.15021155	14	TRBV6-4	TRBJ1-2	CASSDTGVDGYTF	121	0.13865652
15	TRAV8-3	TRAJ34	CAVAISYNTDKLIF	183	0.14391997	15	TRBV3-1	TRBJ2-7	CASSEQGVEQFF	94	0.10771664
16	TRAV12-3	TRAJ37	CAVASGNTGKLIF	168	0.13212325	16	TRBV9	TRBJ1-6	CASSITPGQGSNSPLHF	90	0.10313295
17	TRAV12-3	TRAJ53	CAMRGNSGGSNYKLTF	161	0.12661812	17	TRBV3-1	TRBJ2-7	CASSQGGIEQYF	89	0.10198703
18	RAV38-2/DV	TRAJ43	CALGELVNDMRF	154	0.12111298	18	TRBV30	TRBJ1-2	CAWNEIGGFYGYTF	88	0.10084111
19	TRAV30	TRAJ16	CGHLVDGQKLLF	143	0.11246205	19	TRBV27	TRBJ2-7	CASSQEGIEQYF	86	0.09854926
20	TRAV12-2	TRAJ23	CVVTNQGGKLIF	113	0.08886862	20	TRBV3-1	TRBJ2-7	CASSREGIEQYF	84	0.09625742
21	TRAV12-2	TRAJ23	CAVTNQRGKLIF	112	0.08808217	21	TRBV3-1	TRBJ2-7	GASSQEGIEQYF	80	0.09167373
22	TRAV12-2	TRAJ37	CASKGNTGKLIF	106	0.08336348	22	TRBV3-1	TRBJ2-7	CANSQEGIEQYF	78	0.08938189
23	TRAV12-2	TRAJ37	CAMASGNTGKLIF	97	0.07628545	23	TRBV27	TRBJ2-2	WASSLETGGTGELFF	78	0.08938189
24	TRAV12-2	TRAJ23	CAVTNQGEKLIF	97	0.07628545	24	TRBV3-1	TRBJ2-7	CASSEQGVEQYF	75	0.08594412
25	TRAV1-2	TRAJ23	CAVTNQGGKLIF	97	0.07628545	25	TRBV3-1	TRBJ2-7	WASSQEGIEQYF	72	0.08250636
26	TRAV12-2	TRAJ37	CAVASGNTDKLIF	96	0.075499	26	TRBV3-1	TRBJ2-1	CASSQEGIEQFF	65	0.07448491
27	TRAV12-2	TRAJ37	CAVESGNTGKLIF	95	0.07471255	27	TRBV3-1	TRBJ2-7	CASSQEEIEQYF	63	0.07219306
28	TRAV12-2	TRAJ23	CDVTNQGGKLIF	94	0.07392611	28	TRBV3-1	TRBJ2-2	CASSLETGGTGELFF	63	0.07219306
29	TRAV12-2	TRAJ23	CAVANQGGKLIF	90	0.07078031	29	TRBV3-1	TRBJ2-7	CVSSQEGIEQYF	62	0.07104714
30	TRAV12-3	TRAJ29	CASLRGPLVF	89	0.06999387	30	TRBV3-1	TRBJ2-7	CASRQEGIEQYF	53	0.06073385
31	TRAV12-2	TRAJ23	CAMTNQGGKLIF	84	0.06606163	31	TRBV27	TRBJ2-2	CASSLGTGGTGELFF	53	0.06073385
32	TRAV12-2	TRAJ37	CVVASGNTGKLIF	82	0.06448873	32	TRBV3-1	TRBJ2-7	CASGQEGIEQYF	50	0.05729608
33	TRAV12-2	TRAJ37	CAVASGNAGKLIF	76	0.05977004	33	TRBV3-1	TRBJ2-7	CASNQEGIEQYF	49	0.05615016
34	TRAV38-1	TRAJ34	CAFMISTDKLIF	74	0.05819715	34	TRBV3-1	TRBJ2-7	CARSQEGIEQYF	49	0.05615016
35	TRAV12-2	TRAJ23	CAVNNQGGKLIF	73	0.0574107	35	TRBV3-1	TRBJ2-7	CASSKEGIEQYF	48	0.05500424
36	TRAV6	TRAJ37	CARRRGSGNTGKLIF	68	0.05347846	36	TRBV3-1	TRBJ2-7	CAGSQEGIEQYF	47	0.05385832
37	TRAV12-2	TRAJ29	CASLRGHLVF	64	0.05033267	37	TRBV3-1	TRBJ2-1	WASSEQGVEQFF	45	0.05156647
38	TRAV12-2	TRAJ23	CAVTNQGRKLIF	62	0.04875977	38	TRBV27	TRBJ2-2	CASSVETGGTGELFF	44	0.05042055
39	TRAV20	TRAJ27	CAVREEGQCRQSTF	60	0.04718688	39	TRBV3-1	TRBJ2-1	GASSEQGVEQFF	42	0.04812871
40	TRAV12-2	TRAJ37	CAVVSGNTGKLIF	59	0.04640043	40	TRBV27	TRBJ1-4	CASSTTLYGANEKLFF	42	0.04812871
41	TRAV12-2	TRAJ37	CAVASDNTGKLIF	59	0.04640043	41	TRBV7-8	TRBJ1-2	CASSPPTSGYGYTF	41	0.04698279
42	TRAV12-2	TRAJ29	CASLRGTLVF	58	0.04561398	42	TRBV3-1	TRBJ2-1	CARSEQGVEQFF	41	0.04698279
43	TRAV12-1	TRAJ49	CVVTDPFTGNQFYF	58	0.04561398	43	TRBV3-1	TRBJ2-7	CASSQEGVEQYF	40	0.04583687
44	TRAV12-2	TRAJ37	CAVASGNTGKLIL	57	0.04482753	44	TRBV29-1	TRBJ1-4	CSVGTGHEKLFF	40	0.04583687
45	TRAV36/DV7	TRAJ34	CALATDKLIF	53	0.04168174	45	TRBV3-1	TRBJ2-7	CTSSQEGIEQYF	39	0.04469094
46	TRAV12-2	TRAJ23	CTVTNQGGKLIF	51	0.04010884	46	TRBV3-1	TRBJ2-1	CAGSEQGVEQFF	39	0.04469094
47	TRAV12-2	TRAJ23	GAVTNQGGKLIF	50	0.0393224	47	TRBV7-9	TRBJ2-3	CASSPWGGSDTDTQYF	37	0.0423991
48	TRAV12-2	TRAJ23	CAVTSQGGKLIF	50	0.0393224	48	TRBV27	TRBJ2-1	CASSEQGVEQFF	37	0.0423991
49	TRAV12-2	TRAJ37	WAVASGNTGKLIF	49	0.03853595	49	TRBV3-1	TRBJ2-7	CASSQEGIKQYF	35	0.04010726
50	TRAV12-2	TRAJ23	CAVTNRGGKLIF	49	0.03853595	50	TRBV27	TRBJ2-2	CASSLETGGTEELFF	35	0.04010726

Top 50 of TCR usage and CDR3 sequences with frequency in the library analyzed were summarized.

Supplementary Table 3| Subject information for single cell RNA-sequencing analysis.

Hashtag	Category	ID	dp symptomatic	Cell number
1	moderate	CV-004	46	1510
2			80	546
3			105	357
4			448	90
5		CV-001	122	1089
6			502	204
7		CV-003	82	440
8	severe	CV-037	155	83
9		CV-045	33	53
10		CV-048	63	80

Summary for the subjects, days post symptomatic, and the number of cells whose transcriptomic data was obtained.

Supplementary Table 4| Subject information for single cell RNA-sequencing analysis.

Cytotoxicity	Exhaustion consensus	
CTSW	*TOX*	*ID3*
GNLY	*HAVCR2*	*CCR5*
GZMA	*ENTPD1*	*GOLIM4*
GZMB	*PDCD1*	*ACP5*
GZMH	*CTLA4*	*HLA-DRA*
IFNG	*CD38*	*FCRL3*
KLRB1	*TIGIT*	*OSBPL3*
KLRD1	*VCAM1*	*ICOS*
KLRK1	*CD27*	*FAM3C*
NKG7	*SNAP47*	*PTPN11*
PRF1	*IGFLR1*	*CKS2*
	RAD51	*GALM*
	CCNB1	*SNX9*
	BUB1	*IRF4*
	SIRPG	*STMN1*
	SEMA4A	*PRDM1*
	CXCR6	*CD2BP2*
	FUT8	*RAB27A*
	HLA-DMA	*DUSP4*
	ITGAE	*PHLDA1*
	UBE2F	*ITM2A*
	NDFIP2	*IFI35*
	CD63	*ISG15*
	FKBP1A	*STAT3*
	TPI1	*WARS*
	CDCA8	*SYNGR2*
	NCAPG2	*GBP2*
	CDKN3	*LYST*
	CCL4L2	*BST2*
	RGS2	*PARK7*
	NAB1	

Signature gene lists for cytotoxicity and exhaustion consensus, obtained from public data (see Methods).

Supplementary Table 5| T cell clones with TCRα_{rank1} and TCRβ_{rank1}

clone ID	Hashtag	sub ID	TCRβ					TCRα				
			amino acid	nucleotide	V	J	C	amino acid	nucleotide	V	J	C
14	5, 6	CV-001	**CASSQEGIEQYF**	TGTGCCAGCAGCCAAGAGGGGATCGAGCAGTACTTC	TRBV3-1	TRBJ2-7	TRBC2	**CAVTNQGGKLIF**	TGTGCCGTGACCAACCAGGGAGGAAAGCTTATCTTC	TRAV12-2	TRAJ23	TRAC
(247)	5	CV-001	**CASSQEGIEQYF**	TGTGCCAGCAGCCAAGAGGGGATCGAGCAGTACTTC	TRBV3-1	TRBJ2-7	TRBC2					
58	5, 6	CV-001	**CASSQEGIEQYF**	TGTGCCAGCAGCCAAGAAGGAATCGAGCAGTACTTC	TRBV3-1	TRBJ2-7	TRBC2	**CAVTNQGGKLIF**	TGTGCCGTGACTAACCAGGGAGGAAAGCTTATCTTC	TRAV12-2	TRAJ23	TRAC
(454)	6	CV-001	**CASSQEGIEQYF**	TGTGCCAGCAGCCAAGAAGGAATCGAGCAGTACTTC	TRBV3-1	TRBJ2-7	TRBC2					

T cell clones having TCRα_{rank1} and/or TCRβ_{rank1}, which had been found in the library assay. CDR3 sequences (amino acid and nucleotide), as well as V, J (and C) usage were shown for each clones. Shaded line indicates the clones having no TCRα information.
