# Supplementary material for: Dysfunctional Sars-CoV-2-M protein-specific cytotoxic T lymphocytes in patients recovering from severe COVID-19
Source: Nat Commun. 2022 Dec 16;13:7063. doi: 10.1038/s41467-022-34655-1 (PMC9758236; doi:10.1038/s41467-022-34655-1)
Supplement: Supplementary file 3 — Reporting Summary [file 41467_2022_34655_MOESM3_ESM.pdf]

nature portfolio

Corresponding author(s): Satoshi Ishido, Hideki Ogura

Last updated by author(s): Oct 14, 2022

Reporting Summary

Nature Portfolio wishes to improve the reproducibility of the work that we publish. This form provides structure for consistency and transparency in reporting. For further information on Nature Portfolio policies, see our Editorial Policies and the Editorial Policy Checklist.

Statistics

For all statistical analyses, confirm that the following items are present in the figure legend, table legend, main text, or Methods section.

n/a	Confirmed	
☐	☒	The exact sample size (*n*) for each experimental group/condition, given as a discrete number and unit of measurement
☐	☒	A statement on whether measurements were taken from distinct samples or whether the same sample was measured repeatedly
☐	☒	The statistical test(s) used AND whether they are one- or two-sided<br>*Only common tests should be described solely by name; describe more complex techniques in the Methods section.*
☐	☒	A description of all covariates tested
☐	☒	A description of any assumptions or corrections, such as tests of normality and adjustment for multiple comparisons
☐	☒	A full description of the statistical parameters including central tendency (e.g. means) or other basic estimates (e.g. regression coefficient) AND variation (e.g. standard deviation) or associated estimates of uncertainty (e.g. confidence intervals)
☐	☒	For null hypothesis testing, the test statistic (e.g. *F*, *t*, *r*) with confidence intervals, effect sizes, degrees of freedom and *P* value noted<br>*Give P values as exact values whenever suitable.*
☒	☐	For Bayesian analysis, information on the choice of priors and Markov chain Monte Carlo settings
☒	☐	For hierarchical and complex designs, identification of the appropriate level for tests and full reporting of outcomes
☒	☐	Estimates of effect sizes (e.g. Cohen's *d*, Pearson's *r*), indicating how they were calculated

Our web collection on statistics for biologists contains articles on many of the points above.

Software and code

Policy information about availability of computer code

Data collection	Flow cytometry data were collected by BD FACSDiva V8.0 or MACSQuantify version 2.4.
Data analysis	Flowcytometry data were analyzed with FlowJoTM (v10.4.2) software for Mac OS X and with GraphPad Prism 7.02. scRNA-seq data were analyzed by BBrowser (version 3.3.6).

For manuscripts utilizing custom algorithms or software that are central to the research but not yet described in published literature, software must be made available to editors and reviewers. We strongly encourage code deposition in a community repository (e.g. GitHub). See the Nature Portfolio guidelines for submitting code & software for further information.

Data

Policy information about availability of data

All manuscripts must include a data availability statement. This statement should provide the following information, where applicable:

- Accession codes, unique identifiers, or web links for publicly available datasets
- A description of any restrictions on data availability
- For clinical datasets or third party data, please ensure that the statement adheres to our policy

Immune Epitope Database is accessible online (http://iedb.org). scRNA-seq data on M198-206 specific CD8+ T cells has been deposited in Gene Expression Omnibus datasets under accession no. GSE209676.

Human research participants

Policy information about studies involving human research participants and Sex and Gender in Research.

Reporting on sex and gender	A total of 36 COVID-19 convalescents and 9 healthy volunteers were enrolled in the study, and 20 convalescents and 8 healthy volunteers among them were subjected to the library assay. In addition, 36 convalescents and 6 healthy volunteers were subjected to examination by MHC tetramer. The number of mild, moderate and severe subject were 8, 8 and 4, respectively, for the library assay. Time post-onset, which is given in days since a positive COVID-19 diagnosis was not different between severe and moderate groups. Sex was not considered in the study design and whether sex of participans was determined based on self-report.
Population characteristics	See Supplementary Table 1.
Recruitment	Participants in this study were recruited from Hyogo College of Medicine, hospitals affiliated with Hyogo College of Medicine and Kyowa-kai Medical Corporation based on the agreement with the project. We enrolled all the patients who had written informed consent and therefore there was no self-selection bias or other biases.
Ethics oversight	Ethical approval was given by the ethics committee of Hyogo College of Medicine (reference: 202104-144). All participants were enrolled through informed consent and peripheral blood was drawn by medical doctors under the supervision of the authors.

Note that full information on the approval of the study protocol must also be provided in the manuscript.

Field-specific reporting

Please select the one below that is the best fit for your research. If you are not sure, read the appropriate sections before making your selection.

☒ Life sciences ☐ Behavioural & social sciences ☐ Ecological, evolutionary & environmental sciences

For a reference copy of the document with all sections, see nature.com/documents/nr-reporting-summary-flat.pdf

Life sciences study design

All studies must disclose on these points even when the disclosure is negative.

Sample size	For CD8+ T cell library assay, a total of 20 COVID-19 convalescents and 8 healthy volunteers were enrolled who had HLA-A24*02 allele. Additionally, a total of 36 COVID-19 convalescents and 6 healthy volunteers were enrolled for the tetramer staining analysis. Sample size was determined by the previous work where the similar technique was used (doi:10.4049/jimmunol.1800267). For phenotypic analysis, we had a limitation on samples due to the restricted cell number and had to choose minimum number required for a statistical assessment.
Data exclusions	Two of healthy volunteers and 1 of COVID-19 convalescents enrolled in the library assay had limited number of CD45RO+ CD8+ T cells and/or limited expansion of cells, therefore they were excluded.
Replication	Due to the restricted cell number, T cell library assay and scRNA-seq analysis were no possible. All the other analysis including immunological phenotypes (e.g., cytokine production, expression of cell surface markers) were repeated at least two times. All attempts at replication were successful.
Randomization	No clinical trial nor therapeutic intervention was made in our cohort in this study and randomization was not be appropriate in this study whose aim is exploration of CTLs contributed to recovery.
Blinding	Samples were blinded for T cell library assay or other associated assays including phenotypic analysis. Blinding could not be appropriate for the cytotoxicity assay using SARS-CoV-2 infected cells, because it was conducted under P3 level and minimum number of the samples could be assessed. The moderate samples having had good expansion was chosen in the experiment.

Reporting for specific materials, systems and methods

We require information from authors about some types of materials, experimental systems and methods used in many studies. Here, indicate whether each material, system or method listed is relevant to your study. If you are not sure if a list item applies to your research, read the appropriate section before selecting a response.

Materials & experimental systems

n/a	Involved in the study
☐	☒ Antibodies
☐	☒ Eukaryotic cell lines
☒	☐ Palaeontology and archaeology
☒	☐ Animals and other organisms
☒	☐ Clinical data
☒	☐ Dual use research of concern

Methods

n/a	Involved in the study
☒	☐ ChIP-seq
☐	☒ Flow cytometry
☒	☐ MRI-based neuroimaging

Antibodies

Antibodies used

Flow antibodies;
anti-CD8a-Alexa Fluor 488 (300916, Biolegend, Clone: HIT8a, 1:200)
anti-CD8a-FITC (FITC-65135, Proteintech, Clone: OKT8, 1:50)
anti-CD3-PE (317307, Biolegend, Clone: OKT3, 1:200)
anti-CD4-APC (300552, Biolegend, Clone: RPA-T4, 1:200)
anti-CD45RA-FITC (304148, Biolegend, Clone: HI100, 1:200)
anti-CD45RO-PerCP/Cy5.5 (304222, Biolegend, Clone: UCHL1, 1:200)
anti-PD-1-APC/Cy7 (329921, Biolegend, Clone: EH12.2H7, 1:50)
anti-CD57-PerCP/Cy5.5 (359621, Biolegend, Clone: HNK-1, 1:200)
anti-IFN gamma-APC (502511, Biolegend, Clone: 4S.B3, 1:200)
anti-TNF alpha-BV421 (502931, Biolegend, Clone: MAb11, 1:100)
anti-CCR7-PE (353203, Biolegend, Clone: G043H7, 1:50)
anti-pan HLA (M0736, Dako, Clone: W6/32, 1:200)
control mouse IgG2a (401501, Biolegend, Clone: MG2a-53, 1:1111)
anti-mouse IgG-FITC (406001, Biolegend, 1:200)
anti-TIGIT-PerCp/Cy5.5 (372717, Biolegend, Clone:A15153G,1:200)
Human BD Fc Block (564220, BD Pharmingen, 1:50)
Western blot antibodies;
anti-SARS-CoV-2-S antibody (GTX632604, GeneTex, Clone: 1A9, 1/1,000 in 5% milk TBS-Tween)
anti-SARS-CoV-1-M antibody (AP6008b, Abgent, 1/1,000 in 5% milk TBS-Tween)
anti-SARS-CoV-2-N antibody (GTX135357, GeneTex, 1/1,000 in 5% milk TBS-Tween)
anti-SARS-CoV-2-ORF3a antibody (A20234, ABclonal, 1/1,000 in 5% milk TBS-Tween)
anti-SARS-CoV-2-NSP6 antibody (9177, ProSci, 1/1,000 in 5% milk TBS-Tween)
anti-tubulin (T5168, Sigma-Aldrich, Clone: B-5-1-2, 1/10,000 in 5% milk TBS-Tween)
anti-mouse IgG-HRP (330, MBL, 1/10,000 in 5% milk TBS-Tween)
anti-rabbit IgG (458, MBL, 1/10,000 in 5% milk)

Validation

All the antibodies used in this study are commercially available. Antibodies were appropriately validated by manufacturers and this information is provided on their website and product information datasheets. The antibodies have been further optimized for an appropriate concentration in house.

https://www.biolegend.com/ja-jp/products/alexa-fluor-488-anti-human-cd8a-antibody-3432?Clone=HIT8a (300916, BioLegend, Reactivity: Human, Application: FC)
https://www.ptglab.com/products/CD8a-Antibody-FITC-65135.htm (FITC-65135, Proteintech, Reactivity: Human, Application: FC)
https://www.biolegend.com/ja-jp/products/pe-anti-human-cd3-antibody-36457Clone=OKT3 (317307, Biolegend, Reactivity: Human, Application: FC)

https://www.biolegend.com/ja-jp/products/apc-anti-human-cd4-antibody-823?Clane=RPA-T4 (300552, BioLegend, Reactivity: Human, Application: FC)

https://www.biolegend.com/ja-jp/products/fitc-anti-human-cd45ra-antibody-6867Clone=H1100 (304148, BioLegend, Reactivity: Human, Application: FC)
https://www.biolegend.com/ja-jp/products/percp-cyanine5-5-anti-human-cd45ro-antibody-5604?Clone= UCHL1 (304222, Biolegend, Reactivity: Human, Application: FC) https://www.biolegend.com/ja-jp/products/apc-cyanine7-anti-human-cd279-pd-1-antibody-7121?

Clone=EH12.2H7 (329921, Biolegend, Reactivity: Human, Application: FC)
https://www.biolegend.com/ja-jp/search-results/percp-cyanine5-5-anti-human-cd57-antibody-121277Clone= HNK-1 (359621, Biolegend, Reactivity: Human, Application: FC)

https://www.biolegend.com/ja-jp/products/apc-anti-human-ifn-gamma-antibody-1012?Clane=4S.B3 (502511, Biolegend, Reactivity: Human, Application: FC)

https://www.biolegend.com/ja-jp/search-results/brilliant-violet-421-anti-human-tnf-alpha-antibody-7215?Clone=MAb11 (502931, Biolegend, Reactivity: Human, Application: FC)

https://www.biolegend.com/ja-jp/products/pe-anti-human-cd197-ccr7-antibody-74987Clone=G043H7 (353203, Biolegend, Reactivity: Human, Application: FC)
https://www.labome.com/product/Dako/M0736.html (M0736, Dako, Reactivity: Human, Application: IHC, the same clone was often used for FC by other companies (https://www.biolegend.com/ja-jp/products/purified-anti-human-hla-a-b-c-antibody-1874) and also we confirmed its specificity in HLA-A*24:02 overexpressing cells in FC analysis in the manuscript)

https://www.biolegend.com/ja-jp/products/purified-mouse-igg2a-kappa-isotype-ctrl-2622?Clone=MG2a-53 (401501, BioLegend, Reactivity: KLH, Application: FC, ChIP, WB, IP, IHC) https://www.biolegend.com/ja-jp/products/fitc-anti-mouse-igg-13947Clone=Poly4060 (406001, Biolegend)
https://www.biolegend.com/ja-jp/search-results/percp-cyanine5-5-anti-human-tigit-vstm3-antibody-13948?GroupID=BLG15734 (372717, Biolegend, Reactivity: Human, Application: FC)
https://www.bdbiosciences.com/en-us/products/reagents/flow-cytometry-reagents/research-reagents/single-color-antibodies-ruo/ human-bd-fc-block.564220 (564220, BD Pharmingen, Reactivity: Human, Application: Blocking)
https://www.genetex.com/Product/Detail/SARS-CoV-SARS-CoV-2-COVID-19-spike-antibody-1A9/GTX632604 (GTX632604, GeneTex, Reacivity: SARS-CoV, SARS-CoV-2, Application: WB, ICC/IF, IHC, FC, IP, ELISA) https://www.abeepta.com/products/AP6008b-SARS-Coronavirus-Membrane-Protein-Antibody-C-term (AP6008b, Abgent, Reactivity: SARS-CoV, SARS-CoV-2, the specificity on WB application was confirmed in the overexpression system in the manuscript)
https://www.genetex.com/Product/Detail/SARS-CoV-2-COVID-19-nucleocapsid-antibody/GTX135357 (GTX135357, GeneTex, Reactivity: SARS-CoV, SARS-CoV-2, Aplication: WB, ICC/IF, IHC, IP, ELISA, FC) https://abclonal.co.jp/catalog-antibodies/SARSCoV2ORF3ARabbitpAb/A20234 (A20234, ABclonal, Reactivity: SARS-CoV-2, Application: WB)
https://www.prosci-inc.com/sars-cov-2-covid-19-nsp6-antibody-9177.html (9177, ProSci, Reactivity: Virus, Application: ELISA, the specificity on WB application was confirmed in the overexpression system in the manuscript)
https://www.sigmaaldrich.com/JP/ja/search/t51687

focus=documents&page=1&perPage=30&sort=relevance&term=t5168&type=site_content (T5168, Sigma-Aldrich, Reactivity: various, Application: IF, WB)
https://ruo.mbl.co.jp/bio/dtl/A/7pcd=330 (330, MBL, Reactivity: Mouse, Application: WB, ELISA)
https://ivd.mbl.co.jp/diagnostics/search/detail/?cd=458 (458, MBL, Reactivity: Rabbit, Application: WB, ELISA, IHC)

Eukaryotic cell lines

Policy information about cell lines and Sex and Gender in Research

Cell line source(s)

VeroE6/TMPRSS2 cells (JCRB 1819) were from JCRB, and K562 cells (ATCC CLL-243), phoenix 293T cells (ATCC CRL-3213), Calu-3 cells (ATCC HTB-55) and VeroE6 cells (ATCC CRL-1586) were from ATCC. TG40/CD8a cells were generated by Dr. Ueno, who is a collaborator in this work. 293FT cells were purchased from Invitrogen (R70007).

Authentication

Condition of all employed cell lines were verified by the growth rate and morphology under the microscope. Expression of CD8 alpha on TG40/CD8a was verified by FACS analysis.

Mycoplasma contamination

No mycoplasma contamination was detected.

Commonly misidentified lines (See ICLAC register)	No misidentified cell lines were employed, that was confirmed through ICLA register.

Clinical data

Policy information about clinical studies

All manuscripts should comply with the ICMJE guidelines for publication of clinical research and a completed CONSORT checklist must be included with all submissions.

Clinical trial registration	The approval was given by the ethics committee of Hyogo College of Medicine (reference: 202104-144).
Study protocol	The study protocol is available upon request from authors.
Data collection	The study was performed at Hyogo College of Medicine and samples were collected from adult participants during from October 2020 through March 2022. Clinical data was obtained using routine practice. Immunological data were collected either in real-time (flow cytometry on freshly isolated PBMC samples) or retrospectively (flow cytometry on frozen PBMC samples).
Outcomes	The outcomes reported in this manuscript were a priori defined in the study protocol.<br>Primary outcome was to assess CTL antigenicity against SARS-CoV-2 proteins in COVID-19 convalescents with different severities.<br>Secondary outcome includes functional/phenotypic analysis of SARS-CoV-2 specific CTLs of COVID-19 convalescents.

Flow Cytometry

Plots

Confirm that:

☒ The axis labels state the marker and fluorochrome used (e.g. CD4-FITC).

☒ The axis scales are clearly visible. Include numbers along axes only for bottom left plot of group (a 'group' is an analysis of identical markers).

☒ All plots are contour plots with outliers or pseudocolor plots.

☒ A numerical value for number of cells or percentage (with statistics) is provided.

Methodology

Sample preparation	T cell libraries, TCR-transduced or control TG40/CD8 cells, or cryopreserved PBMCs thawed were stained with PE- and/or BV421-conjugated HLA-A24 M198-206 tetramer, which was prepared in house using QuickSwitch Quant HLA-A*24:02 Tetramer Kit-PE and -BV421 (TB-7032-K1 and TB-73020K4, MBL). Live/Dead Fixable-Near IR or -Aqua staining kit (Invitrogen) was used for eliminating dead cells from the analysis. Next, the cell surface was stained with the listed surface antibodies with appropriate dilutions on ice. Subsequently, the cells were washed and resuspended in 200 ul of 0.5% PFA PBS and kept in dark on ice until flow cytometric acquisition. For some experiments, cells were stimulated with PMA and ionomycin in the presence of brefeldin-A for 4 hours prior to cell surface staining for staining intracellular cytokines IFNg and TNFa with fluorophore-conjugated monoclonal antibodies using FoxP3/transcription factor staining buffer set (eBioscience) according to the manufacturer's instructions.
Instrument	Samples were sorted using BD FACS Aria III (BD Biosciences) and analyzed by either BD LSRFortessa (BD Biosciences) or MACSQuant Analyzer (Miltenyi Biotech).
Software	Data were collected by BD FACSDiva V8.0 or MACSQuantify version 2.4.
Cell population abundance	Sorted samples were checked after being sorted and confirmed high purity (above 99%).
Gating strategy	For CD8+ T cell analysis, cells were first gated on Lymphocyte by forward- and side-scatter gate. Singlets were gated after eliminating dead cells by Live/Dead staining kit.<br>- Tetramer staining of T cell libraries<br>CD8+ PE-tetramer+ cells were identified by comparing with the samples stained with negative control tetramer.<br>- Tetramer staining of PBMCs<br>CD8+ PE-tetramer+ BV421-tetramer+ cells were assessed for the expression of CD45RA/CCR7 and/or PD-1 and CD57. Tetramer positive population was gated based on the samples with negative control tetramer.<br>- ICS of T cell libraries<br>CD8+ IFNg+ TNFa+ cells were identified in M198-206 peptide-stimulated cells. Cytokine positive/negative cells were gated based on solvent control DMSO-treated samples.<br>- ICS of PBMCs<br>CD3+ CD8+ IFNg+ TNFa+ cells were identified in M198-206 peptide stimulated cells. Cytokine positive/negative cells were gated based on solvent control DMSO-treated samples.<br>- Examination of TCR alpha rank1/ beta rank1-reconstituted TG40/CD8 T cells<br>For analysis of TCR alpha rank1/ beta rank1-transduced or control virus-infected TG40/CD8 cells, CD8+ GFP+ were gated. Presence of tetramer positive cells was confirmed by comparing with the samples stained with control tetramer.

☒ Tick this box to confirm that a figure exemplifying the gating strategy is provided in the Supplementary Information.
